# The effect of comorbidities on survival in persons with Alzheimer’s disease: a matched cohort study

**DOI:** 10.1186/s12877-021-02130-z

**Published:** 2021-03-09

**Authors:** Blair Rajamaki, Sirpa Hartikainen, Anna-Maija Tolppanen

**Affiliations:** 1 School of Pharmacy, Faculty of Health Sciences, Kuopio Campus, University of Eastern Finland, P.O. Box 1627, FI-70211 Kuopio, Finland; 2 Kuopio Research Centre of Geriatric Care, University of Eastern Finland, P.O. Box 1627, FI-70211 Kuopio, Finland

**Keywords:** Alzheimer’s disease, Mortality, Survival, Comorbidities, Register studies

## Abstract

**Background:**

Alzheimer’s disease (AD) is one of the leading causes of death world-wide, but little is known on the role of comorbidities on mortality among people with AD. We studied how comorbidities and age at AD diagnosis impact the survival of people with AD.

**Methods:**

The Medication Use and Alzheimer’s disease (MEDALZ) cohort study included 70,718 community-dwelling persons in Finland with AD diagnosis from 2005 to 2011 and were matched 1:1 (age, gender, and hospital district) to people without AD (mean age 80 years, 65% women, and the mean follow-up 4.9 and 5.6 years, respectively). Covariates (age, gender, and socioeconomic position), comorbidities (cardiovascular disease, stroke, diabetes, asthma/ chronic obstructive pulmonary disease (COPD), hip fracture, cancer treatment, and mental or behavioral disorders excluding dementia) and survival data were obtained from nationwide registers. Cox proportional hazard models were used to compare risk of death between people with and without AD.

**Results:**

During the follow-up period a greater proportion of the AD cohort died compared to the non-AD cohort (63% versus 37%)**.** In both cohorts, older age, male gender, lower socioeconomic position, and history of comorbidities were associated with shorter survival and higher risk of death. The associations of comorbidities with survival is weaker in the older age groups and people with AD. Hip fracture (adjusted HR 1.35, 95% CI 1.30–1.41), stroke (1.30, 1.27–1.34), and recent cancer treatment (1.29, 1.26–1.32) had the strongest associations in the AD cohort. Age modified the associations in both cohorts (weaker associations among older people).

**Conclusion:**

Alzheimer’s disease is the major factor affecting survival, but comorbidities further decrease survival also in individuals with Alzheimer’s disease. Therefore, appropriate management of care of these comorbidities might affect not only survival but also the wellbeing of this vulnerable population.

**Supplementary Information:**

The online version contains supplementary material available at 10.1186/s12877-021-02130-z.

## Introduction

Understanding factors that may increase the risk of death after a diagnosis of Alzheimer’s disease (AD) is important for health care providers and health policy makers. AD itself has a strong impact on mortality [1] with estimates of survival following diagnosis ranging from 5 to 10 years [2–4]. Still, sociodemographic characteristics and comorbidities have been associated with risk of death in people with AD and dementia. However, the findings have often been inconsistent. For example, male gender [4–8], lower education level [9], and older age at diagnosis [4–8, 10–12] have been associated with higher risk of death in people with dementia, while these associations have not been observed in other studies [10–14].

Studies on the association between comorbidities and mortality in people with AD also have differing results. An increased risk of mortality with comorbidities, such as cardiovascular disease [6, 11], diabetes [6, 9, 11], and stroke [9] have been reported in people with AD, while other studies have not found increased risk with these comorbidities [5]. In addition, comparisons between studies are complicated by differences in definition of comorbidities, as well as analytic approaches. Only a few studies have reported on individual comorbidities and the use of comorbidity indices has been common. These studies have reported increased risk of death in people with dementia with a higher comorbidity burden [15–17].

Most chronic diseases are associated with increased mortality [18–20], but information on the role of comorbidities on survival of people with AD is sparse and conflicting. In addition, the incidence of AD increases with age [21], but to our knowledge there are no studies that have assessed how age modifies the association of comorbidities.

We evaluated how comorbidities at the time of AD diagnosis affect survival and whether the associations are modified by age using data from a nationwide cohort of people with a clinically confirmed AD diagnosis. We also assessed whether the associations were similar in a matched comparison cohort without AD.

## Methods

### Study cohort

The Medication and Alzheimer’s disease (MEDALZ) study is a retrospective matched-cohort study of community-dwelling persons who received a new clinically verified diagnosis of AD from 2005 to 2011 (*N* = 70,718) in Finland. Those with a clinically verified AD diagnosis were identified from the Finnish Special Reimbursement Register (FSRR), which is maintained by the Social Insurance Institution of Finland (SII) as described in a previous article [22]. The FSRR contains records of all people who are eligible for higher reimbursement of medications due to certain chronic diseases, such as AD. For a person to be eligible for the FSRR for AD they need a verified diagnosis of AD written in a medical statement by their physician and submitted to SII. The medical statement must include that the patient has: 1) symptoms consistent with AD, 2) experienced a decrease in social capacity over a period of at least 3 months, 3) received a computed tomography (CT)/ magnetic resonance imaging scan (MRI) to confirm that neuroanatomical changes are consistent with AD, 4) had possible alternative diagnoses excluded, and 5) received confirmation of the diagnosis by a registered neurologist or geriatrician. Along with the medical statement submitted by the physicians to SII, findings from the CT/MRI, laboratory tests, cognitive tests, and statements from the patient and their family are included. Each case is systematically reviewed by a geriatrician/ neurologist to confirm whether pre-specified criteria are met. The AD diagnosis was based mainly on the National Institute of Neurological and Communicative Disorders and Stroke and the Alzheimer’s Disease and Related Disorders Association’s (NINCDS-ADRDA) [23] and Diagnostic and Statistical Manual of Mental Disorders, 4th edition (DSM-IV) [24] criteria for Alzheimer’s disease. People with AD were matched to people without AD based on age (± 1 year), gender, and hospital district region.

Data from the national registers was compiled using the unique personal identity codes assigned to every resident of Finland, which has been previously described [25] and was de-identified by the register maintainers before being released to the research team. Ethics committee approval or informed consent were not required according to the Finnish legislation (Personal Data Act) because only de-identified, routinely collected register data was used and the study participants were not contacted. The MEDALZ study protocol was approved by the register maintainers (Statistics Finland, SII, and National Institute of Health and Welfare) and the University of Eastern Finland.

### Mortality

Mortality data (2005–2015) was obtained from the Causes of Death Register, maintained by Statistics Finland (SF). Persons who entered the FSRR for AD starting on January 1, 2005 (or matching date) and ended on December 31, 2011 were followed until death or end of the follow-up period (December 31, 2015). Maximum follow-up time was 11 years. In addition, people in the non-AD cohort were censored if they were added to the FSRR for AD during follow-up.

### Sociodemographic factors and comorbidities

Socioeconomic position (SEP), defined by the occupational social class, was obtained from the census maintained by Statistics Finland. The 2010 version of the original classification is available from reference [26]. Data on the comorbidities (cardiovascular disease, coronary artery disease, stroke, diabetes, asthma/ chronic obstructive pulmonary disease (COPD), hip fracture, cancer treatment, and mental or behavioral disorders excluding dementia) was gathered from the National Hospital Discharge Register, FSRR, SF, and the Prescription Register, which is also maintained by SII. The definition, measurement period, and data source for each covariate is described in detail in Supplemental Table 1.

### Statistical analyses

Characteristics between AD and non-AD cohorts were compared using t-test for continuous variables and chi-square test for categorical variables. We did two analyses for mortality: one comparing survival time of people with and without AD, and the other comparing risk of death for people with and without AD using Cox proportional hazard models and unadjusted Kaplan-Meier curves. We also compared the risk of mortality by two age categories (< 80 years and ≥ 80 years) according to median age. Proportionality of hazards was confirmed with Kaplan-Meier curves. Mortality risk factor analyses were calculated with the Cox proportional hazard model and were adjusted for age, gender, and SEP when appropriate. Results were expressed as hazard ratios (HRs) with 95% confidence intervals (CI). Summary statistics of incidence rates per 100-person years and survival time by age categories were based on the survival time counted in calendar days and expressed as person years from the start of follow-up to the day of death or to the end of the follow-up. We calculated the risk of mortality with the interaction between age and other covariates also using Cox proportional hazard models. All statistical analyses were performed with STATA/MP 14.2 (StataCorp, College Station, TX, USA).

## Results

### Study sample

Each cohort consisted of 70,718 persons, 65% of whom were women. The mean age of the study sample was 80 years (SD 7.1) (Table 1). In the AD cohort, 44,585 (63.1%) died during the follow-up compared to 26,410 (37.4%) in the non-AD cohort. There were slight differences in the distribution of occupational social class between cohorts with no consistent pattern. Coronary artery disease, stroke, diabetes, a history of hip fracture, recent cancer treatment, and mental or behavioral disorders (excluding dementia) were more prevalent in the AD cohort, while any cardiovascular disease and asthma/COPD had similar prevalence in both cohorts. The mean follow-up time was 8 months shorter in the AD cohort than the non-AD cohort.

**Table 1:** Characteristics of AD and non-AD cohorts

Characteristic	AD Cohort N = 70,718	non-AD Cohort N = 70,718	*P*-value
**Sociodemographic factors**			
Age at beginning of follow-up, mean (SD)	80.05 (7.1)	80.02 (7.1)	matched
Categorized age at beginning of follow-up			0.429
< 80 y	31,971 (45.21)	32,119 (45.42)	
≥ 80 y	38,747 (54.79)	38,599 (54.58)	
Sex			matched
Women	46,116 (65.21)	46,116 (65.21)	
Men	24,602 (34.79)	24,602 (34.79)	
Highest occupational social class			< 0.001
Managerial/ professional	14,692 (20.78)	14,929 (21.11)	
Office worker	5973 (8.45)	5888 (8.33)	
Farming/forestry	13,439 (19.00)	13,990 (19.78)	
Sales/industry/cleaning	30,148 (42.63)	27,748 (39.24)	
Unknown/did not respond	6466 (9.14)	8163 (11.54)	
**Comorbidities**			
Any cardiovascular disease	35,026 (49.53)	34,757 (49.15)	0.153
Coronary artery disease	21,668 (30.64)	20,006 (28.29)	< 0.001
Stroke	6825 (9.65)	5833 (8.25)	< 0.001
Diabetes	13,714 (19.39)	8081 (11.43)	< 0.001
Asthma/COPD	7557 (10.69)	7646 (10.81)	0.445
History of hip fracture	3709 (5.24)	2561 (3.62)	< 0.001
Cancer treatment within 5 years of start of follow-up	16,200 (22.91)	12,916 (18.26)	< 0.001
Any mental or behavioral disorder (exc. dementia)	41,106 (58.13)	36,997 (52.32)	< 0.001
**Follow-up**			
Duration of follow-up days, mean (min.-max.)	1787 (7–4016)	2038 (4–4016)	< 0.001
Died during follow-up	44,585 (63.05)	26,410 (37.35)	< 0.001
Died during 4-y follow-up	24,277 (34.33)	15,685 (22.18)	< 0.001

Data are given as n (%) unless otherwise indicated.

Abbreviations: AD, Alzheimer’s disease; COPD, chronic obstructive pulmonary disease; exc., excluding; max., maximum; min., minimum; SD, standard deviation; y, years

During the maximum follow-up, people with AD had 2-fold higher risk of death in comparison to people without AD after adjusting for age, sex, and SEP (adjusted HR 2.04, 95% CI 2.01–2.07). Limiting the follow-up time to four years (the follow-up available for all participants) attenuated this association (adjusted HR 1.62 (95% CI 1.58–1.65).

### Associations between risk factors and survival

In both cohorts, older age, male gender, lower occupational social class and history of comorbidities were associated with shorter survival (Table 2) and higher risk of death (Table 3). The associations were stronger in the non-AD cohort. For example, the relative risk of death associated with age above median was 2.1 (95% CI 2.06–2.14) in people with AD when adjusted for sex and SEP, but a much higher risk increase was seen in people without AD (adjusted HR 3.11, 95% CI 3.02–3.19). Sex was the only characteristic which had a stronger association in the AD cohort: men in the AD cohort had 1.58-fold risk of death (95% CI 1.55–1.62) in comparison to women, while a smaller relative risk increase was found in the non-AD cohort (HR 1.41, 95% CI 1.37–1.45). However, it should be noted that the mortality rates were higher in people with AD than without AD in all categories, i.e. different age- sex- and SEP-strata.

**Table 2:** Incidence rate and median survival times according to covariates

		AD cohort		Non-AD cohort	
	**Category**	**IR/ 100 Person-years**	**Median** survival time in years (min.-max.)	**IR/ 100 Person-years**	**Median** survival time in years (min.-max.)
**Sociodemographic factors**					
Age Category	< 80 y	9.22	5.50 (0.02–11)	3.47	6.33 (0.08–11)
	≥80 y	16.80	4.33 (0.02–11)	10.22	4.84 (0.01–11)
Sex	Women	11.81	5 (0.02–11)	6.36	5.61 (0.01–11)
	Men	15.15	4.5 (0.02–11)	7.35	5.33 (0.03–11)
SEP	Managerial/professional	11.33	4.93 (0.04–11)	4.75	5.75 (0.08–11)
	Office worker	11.75	4.99 (0.02–11)	5.64	5.67 (0.01–11)
	Farming/forestry	13.85	4.72 (0.03–11)	8.21	5.33 (0.06–11)
	Sales/industry/cleaning	12.99	4.78 (0.02–11)	6.94	5.45 (0.02–11)
	Unknown/ Did not respond	15.24	4.56 (0.04–11)	7.85	5.5 (0.08–11)
**Comorbidities**					
Any cardiovascular disease	No	11.24	5.03 (0.02–11)	5.11	5.83 (0.01–11)
	Yes	14.75	4.58 (0.02–11)	8.53	5.16 (0.03–11)
Coronary artery disease	No	11.66	5 (0.02–11)	5.52	5.75 (0.01–11)
	Yes	16.1	4.36 (0.02–11)	10.23	4.89 (0.03–11)
Stroke	No	12.51	4.85 (0.02–11)	6.24	5.6 (0.01–11)
	Yes	16.90	4.25 (0.02–11)	12.96	4.54 (0.02–11)
Diabetes	No	12.46	4.9 (0.02–11)	6.32	5.6 (0.01–11)
	Yes	14.82	4.43 (0.02–11)	10.07	4.84 (0.03–11)
Asthma/COPD	No	12.63	4.84 (0.02–11)	6.4	5.58 (0.02–11)
	yes	15.2	4.43 (0.05–11)	9.43	5 (0.08–11)
History of hip fracture	No	12.63	4.84 (0.02–10.91)	6.65	5.58 (0.01–11)
	Yes	18.49	4.08 (0.02–10.91)	14.88	4.25 (0.06–11)
Cancer treatment within 5 years of start of follow-up	No	12.2	4.91 (0.02–11)	5.6	5.67 (0.01–11)
	Yes	16.37	4.34 (0.02–11)	10.93	4.9 (0.02–11)
Any mental or behavioral disorder (exc. dementia)	No	12.28	4.91 (0.02–11)	5.60	5.75 (0.01–11)
	Yes	13.33	4.75 (0.02–11)	7.77	5.30 (0.02–11)

Abbreviations: AD, Alzheimer’s disease; COPD, chronic obstructive pulmonary disease; exc., excluding; IR, incidence rate; max., maximum; min., minimum; SEP, socioeconomic position; y, years

**Table 3:** Mortality risk factors in the AD and non-AD cohorts

	AD cohort		Non-AD cohort	
	Unadjusted HR (95% CI)	Adjusted HR^**a**^ (95% CI)	Unadjusted HR (95% CI)	Adjusted HR^**a**^ (95% CI)
**Sociodemographic factors**				
Age at baseline, increase per year	1.07 (1.07–1.07)	1.07 (1.07–1.07)	1.11 (1.11–1.11)	1.12 (1.12–1.12)
Age Category				
< 80 y	1.00	1.00	1.00	1.00
≥ 80 y	2.03 (1.99–2.07)	2.10 (2.06–2.14)	3.07 (2.99–3.15)	3.11 (3.02–3.19)
Gender, Men (vs women)	1.34 (1.31–1.36)	1.58 (1.55–1.62)	1.16 (1.13–1.19)	1.41 (1.37–1.45)
Socioeconomic position				
Managerial/professional	1.00	1.00	1.00	1.00
Office worker	1.03 (0.99–1.08)	1.09 (1.05–1.14)	1.19 (1.13–1.26)	1.12 (1.06–1.18)
Farming/forestry	1.23 (1.20–1.27)	1.05 (1.02–1.08)	1.73 (1.67–1.80)	1.29 (1.24–1.34)
Sales/industry/cleaning	1.15 (1.12–1.18)	1.08 (1.05–1.11)	1.46 (1.41–1.52)	1.24 (1.19–1.28)
Unknown/ Did not respond	1.37 (1.32–1.42)	1.16 (1.22–1.21)	1.65 (1.58–1.73)	0.91 (0.87–0.95)
**Comorbidities** (present vs absent)				
Any cardiovascular disease	1.36 (1.33–1.38)	1.25 (1.22–1.27)	1.68 (1.64–1.73)	1.52 (1.49–1.56)
Coronary artery disease	1.45 (1.42–1.48)	1.26 (1.24–1.29)	1.88 (1.83–1.93)	1.57 (1.53–1.61)
Stroke	1.42 (1.38–1.46)	1.30 (1.27–1.34)	2.12 (2.04–2.2)	1.79 (1.73–1.86)
Diabetes	1.23 (1.21–1.26)	1.25 (1.22–1.28)	1.62 (1.56–1.67)	1.66 (1.61–1.72)
Asthma/COPD	1.24 (1.21–1.28)	1.23 (1.19–1.27)	1.49 (1.44–1.54)	1.50 (1.44–1.55)
History of hip fracture	1.57 (1.51–1.63)	1.35 (1.30–1.41)	2.36 (2.24–2.48)	1.71 (1.62–1.8)
Cancer treatment within 5 years of start of follow-up	1.40 (1.37–1.44)	1.29 (1.26–1.32)	1.97 (1.92–2.02)	1.90 (1.85–1.95)
Any mental or behavioral disorder (exc. dementia)	1.09 (1.07–1.11)	1.11 (1.09–1.13)	1.39 (1.36–1.43)	1.41 (1.37–1.44)

Abbreviations: AD, Alzheimer’s disease; CI, confidence interval; COPD, Chronic obstructive pulmonary disease; exc., excluding; HR, hazard ratio; y, years

^a^ Adjusted for age sex and Socioeconomic position when appropriate

Similar patterns were observed with baseline comorbidities (weaker associations indicated by HRs, but shorter median survival times and higher mortality rates in the AD cohort) (Tables 2-3).

### Interaction of covariates and age

The associations between comorbidities and mortality were modified by age (P for interaction < 0.05 for all comorbidities in the adjusted models). When the analyses were stratified by the median age (80 years) comorbidities were still associated with higher risk of death in both age categories in both the AD and non-AD cohorts (Table 4), but stronger associations were observed in the younger age category. Lower relative risks were seen in both age categories in the AD cohort compared to the non-AD cohort.

**Table 4:** Adjusted ^a^ hazard ratio (95% CI) for death by age category ^b^ and the interaction of comorbidities.

	AD cohort		Non-AD cohort	
Comorbidities	< 80 years	≥80 years	< 80 years	≥80 years
Any cardiovascular disease	1.32 (1.28–1.36)	1.25 (1.22–1.28)	1.60 (1.53–1.68)	1.52 (1.47–1.56)
Coronary artery disease	1.36 (1.32–1.41)	1.28 (1.25–1.31)	1.78 (1.69–1.87)	1.59 (1.54–1.64)
Stroke	1.45 (1.38–1.52)	1.25 (1.20–1.30)	2.27 (2.11–2.44)	1.74 (1.68–1.82)
Diabetes	1.29 (1.24–1.34)	1.19 (1.16–1.23)	1.86 (1.75–1.97)	1.52 (1.46–1.58)
Asthma/COPD	1.28 (1.22–1.34)	1.21 (1.17–1.26)	1.71 (1.61–1.83)	1.40 (1.35–1.46)
History of hip fracture	1.64 (1.51–1.79)	1.38 (1.32–1.44)	2.38 (2.09–2.72)	1.87 (1.77–1.97)
Cancer within 5 years of start of follow-up	1.53 (1.48–1.58)	1.23 (1.19–1.27)	2.94 (2.81–3.09)	1.61 (1.56–1.66)
Any mental or behavioral disorder (exc. dementia)	1.11 (1.07–1.14)	1.11 (1.09–1.39)	1.40 (1.33–1.46)	1.42 (1.38–1.46)

Data presented as hazard ratios (95% CI).

^a^ Adjusted for sex and socioeconomic position

^b^ age category at the start of follow-up

Abbreviations: AD, Alzheimer’s disease; CI, confidence interval; COPD, chronic obstructive pulmonary disease; exc., excluding

## Discussion

Our results show that comorbidities at the time of AD diagnosis are associated with decreased survival in people with AD, and the associations are stronger among those with younger age at AD diagnosis. The associations between comorbidities were weaker in people with AD than without AD, which is likely due to the strong impact of AD on mortality [27–30]. Similarly, the weaker associations of comorbidities in the older age group imply that age, together with AD were the main predictors of survival in this study. However, regardless of AD and age, all comorbidities were still associated with higher risk of death*,* i.e.*,* they were associated with lower survival also in people with AD.

### Comorbidities

In 2010, the leading cause of death of people aged 65 years or older were diseases of the circulatory system, including coronary artery disease and stroke, both in Finland [31] and globally [32]. Cardiovascular disease was the second most prevalent comorbidity in our study. In a systematic review and meta-analysis of people with dementia by van de Vorst et al. [33] coronary artery disease and chronic heart failure were associated with mortality. A recent Canadian study of community dwelling people at the time of dementia diagnosis also reported increased risk of death with chronic heart failure, but did not report on other cardiac diseases [8]. However, A Dutch study restricted to people with AD found no association of history of cardiovascular disease (including stroke) and mortality, [7] but the lack of association may be due to small sample size. We found a 5.4-month shorter median survival time of people with cardiovascular disease in the AD cohort and an 8-month shorter survival time of people with cardiovascular disease in the non-AD cohort.

Stroke is an independent risk factor for AD [34], but there is conflicting evidence if a previous stroke is associated with an increased risk of mortality in this population. Steenland et al. [9] found an increased risk in community-dwelling people with AD, while Rountree et al. [5], consistent with the findings from van de Vorst et al. meta-analysis [33], reported no association. The prevalence of history of stroke was less than 10% of our study population but was associated with one of the highest mortality rates in both cohorts. Huyer et al. [8] reported an increased risk of death in community-dwelling people with dementia. We found a 7.2-month shorter median survival time of people with a history of stroke in the AD cohort and a 12.7-month shorter survival time of people with the comorbidity in the non-AD cohort.

Diabetes is also a known risk factor for AD [35]. The meta-analysis by van de Vorst et al. [33] reported an almost 50% greater risk of death in people with diabetes and dementia which was much higher than our results. Studies reporting on people with AD again have conflicting findings on diabetes and the risk of mortality. Steenland et al. [9] reported an increase in risk of mortality with diabetes on community-dwelling people with AD, but Rountree et al. [5] did not find any association. An increased risk of death was also reported in community-dwelling people with diabetes and dementia [8]. We found a 5.6-month shorter median survival time of people with diabetes in the AD cohort and a 9.1-month shorter survival time in the non-AD cohort. A study from Australia of community-dwelling people with AD found a much larger difference, 1.3 years, in median survival time of people with diabetes compared to those without diabetes [36]. The median survival time of people with AD and diabetes was much longer (4.43 years) in our study compared to the Australian study (0.86 years) although both studies are based on time of diagnosis and mean age at AD diagnosis was approximately 80 years.

Respiratory diseases have rarely been studied as a predictor of survival in person with AD or dementia. In a Canadian cohort study of people who were community-dwelling at the time of dementia diagnosis, COPD was associated with an increased risk of mortality (OR 1.7, 95% CI 1.7–1.8) [8]. However, in our study asthma/COPD had one of the weakest associations with mortality in both cohorts, but the risks were still statistically significant. There was also minimal difference in risk of mortality between the two age groups in the AD cohort. The difference in our results compared to the Canadian study may be due to difference in study populations. The prevalence of COPD was slightly higher in the Canadian study compared to our study (14.8% versus 10.7% in the AD cohort). In our study, initiation of anti-dementia drugs according to Finnish guidelines of care was planned. People with AD and severe asthma/COPD may not be able to use acetylcholinesterase inhibitors due to possible worsening of asthma/ COPD and therefore they would not have been captured in our study population. Asthma and COPD are part of Comorbidity Indices [37, 38] used in several studies [16, 17], but by reporting comorbidities in this way makes it impossible to see an association between individual comorbidities and mortality. We found a 4.9-month shorter median survival time of people with a history of asthma/COPD in the AD cohort and a 7-month shorter survival time in the non-AD cohort. To the best of our knowledge, no other studies have reported on the survival time of people with AD and chronic respiratory diseases.

The prevalence of cancer and AD both increase with increasing age [39], but few studies have looked at the impact a history of cancer has on the risk of mortality of people with AD or dementia. An inverse relationship of developing AD after surviving cancer has been reported [40, 41], however, a recent history of cancer treatment was more prevalent in the AD cohort compared to the non-AD cohort. We limited the cancer diagnosis to treatment within 5 years of start of follow-up because we wanted to restrict the analysis to recent history of cancer. In our study the risk of mortality increased with a history of cancer in both cohorts. Helzner et al. [11] also reported an increased risk of mortality in people with AD and a history of cancer, but they were not statistically significant. Studies on people with dementia have reported an increased risk of mortality for people with a history of cancer [8]. Recent cancer treatment had the greatest difference between the younger and older groups. We found a 6.8-month shorter median survival time of people with a history of recent cancer treatment in the AD cohort and a 9.2-month shorter survival time of people with the comorbidity in the non-AD cohort. To the best of our knowledge, no other studies have reported on the survival time of people with AD and history of cancer.

Previous studies have reported on the increase in risk of mortality after incident hip fracture in people with AD [42, 43], but none have looked at how a history of fractures prior to AD diagnosis affects survival. We found a history of hip fracture to have the highest relative risk of mortality for people with AD. Age modified the association of hip fracture and mortality, with greater risk of death seen in the younger age group. We found a 9.1-month shorter median survival time of people with a history of hip fracture in the AD cohort and a 16-month shorter survival time of people with the comorbidity in the non-AD cohort. Again, most studies have reported on survival time of people with AD after incident hip fracture, so we found no other studies for comparison.

Incidence of mental and behavioral disorders is elevated prior to an AD diagnosis [44] as prodromal signs and symptoms of AD may be diagnosed as mental and behavioral disorders [39]. Larson et al. [6] found baseline psychiatric symptoms or behavioral disturbances were not strongly associated with survival in people with AD. Because severe mental disorders, including schizophrenia and severe depression, have a large negative impact on life expectancy [45], a considerable proportion of people with these severe disorders do not survive to the typical onset of AD. This is a likely explanation for the weaker association between mental and behavioral disorders and survival after AD diagnosis in comparison to the other comorbidities. We found a 2-month shorter median survival time of people with a history of mental or behavioral disorder in the AD cohort and a 5.4-month shorter survival time of people with the comorbidity in the non-AD cohort. Again, to the best of our knowledge no other studies were found for comparison.

### Demographic factors

The associations of sociodemographic characteristics and mortality were comparable to previous studies, supporting the external validity of our results. Previous studies have reported the risk of mortality increases 4–7% per one year of age [3]. The association between comorbidities and mortality was weaker in the older age groups and this was observed in both groups (people with and without AD). This might be due to survivor bias, i.e. those aged over 80 years are a minor part of their birth cohort and have survived despite the comorbidities [46].

The higher risk of death in men has also consistently been reported in previous studies [3]. Higher SEP, indicated by higher occupational social class was associated with lower risk of death in our study. In previous studies of people with dementia, education level has commonly been used to represent SEP and the findings have been heterogeneous [14]. Steenland et al. [9] observed lower mortality among those with higher level of education while other studies found association between lower level of education and lower risk of death [47, 48], or observed no difference [4, 5, 7, 11, 14, 15]. SEP is notoriously difficult to capture, and different indicators may not be unanimous. Thus, differences in SEP indicators, in addition to between- country differences may explain the results.

Strengths of our study include a nationwide cohort of people with clinically verified AD diagnosis in Finland. Studies assessing the internal validity of the Finnish Care for Health care and comparing register information with patient records or other information from the primary source have confirmed that the coverage and accuracy of these registers are well suited for epidemiological research [49, 50]. Through the study design we did not have loss to follow-up. Only a few studies have reported on the individual comorbidities like we have, and none have reported on the survival times in a comparable manner.

Unfortunately we do not have information on the stage or severity of AD at diagnosis, which has been associated to mortality [3]. The survival times may be overestimated (survivorship bias) since our study design was unable to capture those with rapidly progressive disease that died before getting diagnosed with AD. The follow-up time which was on average 4.9 years for the AD cohort, but a proportion of the cohort had shorter follow-up time due to the study design.

## Conclusion

Alzheimer’s disease is the major factor affecting survival, but comorbidities further decrease survival in individuals with Alzheimer’s disease. Therefore, appropriate management of care of these comorbidities might affect not only survival but also the wellbeing of this vulnerable population.

## Supplementary Information

**Additional file 1.**

## Data Availability

The data that support the findings of this study are available from the Social Insurance Institution (SII) but restrictions apply to the availability of these data, which were used under license for the current study, and so are not publicly available. Data are however available from the authors (contact A-MT) upon reasonable request and with permission of SII.

## Abbreviations

AD: Alzheimer’s disease
CI: Confidence Interval
COPD: Chronic obstructive pulmonary disease
CT: Computed tomography
FSRR: Finnish Special Reimbursement Register
HR: Hazard Ratio
MEDALZ: Medication use and Alzheimer’s disease study
MRI: Magnetic resonance imaging scan
SEP: Socioeconomic position
SF: Statistics Finland
SII: Social Insurance Institution of Finland
