# Supplementary material for: The effect of comorbidities on survival in persons with Alzheimer’s disease: a matched cohort study
Source: BMC Geriatr. 2021 Mar 9;21:173. doi: 10.1186/s12877-021-02130-z (PMC7941944; doi:10.1186/s12877-021-02130-z)
Supplement: Supplementary file 1 — Additional file 1. [file 12877_2021_2130_MOESM1_ESM.docx]

Supplementary Table

The effect of comorbidities on survival in persons with Alzheimer’s disease: a matched cohort study

Blair Rajamaki (MSc), Sirpa Hartikainen (MD, PhD), Anna-Maija Tolppanen (PhD)

| **Supplemental Table 1** Definition for covariates | | | |
| --- | --- | --- | --- |
|  | **ICD-code/ ATC code/ other code** | **Measurement period** | **Data sources** |
| **Sociodemographic/socioeconomic factors** | | | |
| Age, years | <80  ≥80 | Start of follow-up | SII |
| Sex | Male  Female | Start of follow-up | SII |
| Occupational socioeconomic class | Managerial/ professional  Officer worker  Farming/forestry  Sales/industry/cleaning  Unknown/no response | The highest position recorded since 1972 until the start of follow-up | SR |
| **Comorbidities** | | | |
| Any cardiovascular disease (hypertension, coronary artery disease, familial hypercholesterolemia, heart failure, and chronic cardiac arrhythmias) | special reimbursement (classification numbers 201, 205, 206, 213, 280) | Diagnosed since 1972 until the start of follow-up | SRR |
| Coronary artery disease | ICD-10: I20-I25 (hospital discharge register) | Diagnosed since 1996 until the start of follow-up | HDR |
|  | special reimbursement (classification numbers 206) | Diagnosed since 1972 until the start of follow-up | SRR |
| Stroke | ICD10: I60-I64 (hospital discharge register) | Diagnosed since 1996 until the start of follow-up | HDR |
| Diabetes | Diabetes medication (ATC: A10, excluding A10BX01 guar gum) | At least one purchase prior to the start of follow-up in the prescription register (1995-follow-up) | PR |
|  | special reimbursement for diabetes (classification number 103) | Diagnosed since 1972 until the start of follow-up | SRR |
| Asthma/ COPD | ICD-10: J44-J46 (hospital discharge register) | Diagnosed since hospitalization 1996 | HDR |
|  | special reimbursement (classification number 203) | Diagnosed since 1972 until the start of follow-up | SRR |
| Hip fracture | ICD-10: S72.0-S72.2 (hospital discharge register) | Diagnosed since 1996 until the start of follow-up | HDR |
| Cancer treatment | ICD10: C00-C97 (hospital discharge register) | Diagnosed since 1996 until the start of follow-up | HDR |
|  | ATC: L01 (excluding persons with L01BA01 and special reimbursement for rheumatoid arthritis), L02, L03AA, L03AB01, L03AB04, L03AB05, L03AC, L03AX (excluding L03AX13), L04AA10, L04AA34, L04AA18, L04AX02, L04AX03 (excluding persons with special reimbursement for rheumatoid arthritis) | Within five years prior to the start of follow-up | PR |
| Any mental or behavioral disorder (excluding dementia) | ICD-10: F* codes (excluding dementia; hospital discharge register)) | Diagnosed since 1996 until the start of follow-up | HDR |
|  | special reimbursement (classification number 112) | Diagnosed since 1972 until the start of follow-up | SRR |
| Start of follow-up is the date of Alzheimer’s disease diagnosis or corresponding matching date for the non-users.  Abbreviations: ATC, Anatomical Therapeutic Chemical; HDR, Hospital Discharge Register; ICD, International Classification of Diseases; NOMESCO, Nordic Medico-Statistical Committee; PR, Prescription Register; SF, Statistics Finland; SII, Social Insurance Institution of Finland; SRR, Special Reimbursement Register. | | | |
